# Overexpression of Brain-Derived Neurotrophic Factor Protects Large Retinal Ganglion Cells After Optic Nerve Crush in Mice

**DOI:** 10.1523/ENEURO.0331-16.2016

**Published:** 2017-01-17

**Authors:** Liang Feng, Zhen Puyang, Hui Chen, Peiji Liang, John B. Troy, Xiaorong Liu

**Affiliations:** 1 Department of Ophthalmology, Feinberg School of Medicine, Northwestern University, Chicago, IL 60611, USA; 2 Department of Neurobiology, Weinberg College of Arts and Sciences, Northwestern University, Evanston, IL 60208, USA; 3 Department of Biomedical Engineering, Robert R. McCormick School of Engineering and Applied Science, Northwestern University, Evanston, IL 60208, USA; 4 School of Biomedical Engineering, Shanghai Jiao Tong University, Shanghai 200240, China

**Keywords:** brain-derived neurotrophic factor (BDNF), in vivo imaging, neuroprotection, optic nerve crush, retinal ganglion cells (RGCs)

## Abstract

Brain-derived neurotrophic factor (BDNF), a neurotrophin essential for neuron survival and function, plays an important role in neuroprotection during neurodegenerative diseases. In this study, we examined whether a modest increase of retinal BDNF promotes retinal ganglion cell (RGC) survival after acute injury of the optic nerve in mice. We adopted an inducible Cre-recombinase transgenic system to up-regulate BDNF in the mouse retina and then examined RGC survival after optic nerve crush by *in vivo* imaging. We focused on one subtype of RGC with large soma expressing yellow fluorescent protein transgene that accounts for ∼11% of the total SMI-32–positive RGCs. The median survival time of this subgroup of SMI-32 cells was 1 week after nerve injury in control mice but 2 weeks when BDNF was up-regulated. Interestingly, we found that the survival time for RGCs taken as a whole was 2 weeks, suggesting that these large-soma RGCs are especially vulnerable to optic nerve crush injury. We also studied changes in axon number using confocal imaging, confirming first the progressive loss reported previously for wild-type mice and demonstrating that BDNF up-regulation extended axon survival. Together, our results demonstrate that the time course of RGC loss induced by optic nerve injury is type specific and that overexpression of BDNF prolongs the survival of one subgroup of SMI-32–positive RGCs.

## Significance Statement

The primary goal of this study was to investigate the role of BDNF on RGC survival after optic nerve injury. We adopted an inducible Cre-recombinase transgenic system to modestly up-regulate BDNF in the mouse retina. We then applied a live imaging technique to track the survival of RGCs expressing yellow fluorescent protein *in vivo*. We identified one type of RGC with a large soma that accounts for ∼11% of the total SMI-32–positive RGCs. Our results showed that these SMI-32-positive large-soma cells were susceptible to acute optic nerve injury. Furthermore, we found that BDNF up-regulation promoted survival of these large-soma SMI-32–positive RGCs. Our study thus adds new insights on better understanding of type-specific RGC loss after optic nerve injury and the underlying BDNF-mediated neuroprotective mechanism.

## Introduction

Optic neuropathy refers to optic nerve injury resulting in optic atrophy and retinal ganglion cell (RGC) dysfunction, induced by insults such as glaucoma or acute damage to the optic nerve. Different animal models of experimental optic neuropathy have been created to examine RGC degeneration and its underlying mechanisms (Morrison et al., 2011; Calkins, 2012; Puyang et al., 2015). Recent studies, including ours, suggest that RGCs degenerate in a type-specific order (Della Santina et al., 2013; Feng et al., 2013; Chen et al., 2015; Duan et al., 2015; El-Danaf and Huberman, 2015; Ou et al., 2016). One study reported that OFF arbors of all RGC subtypes were damaged by a transient elevation of intraocular pressure (IOP; El-Danaf and Huberman, 2015), whereas we have found that both OFF RGCs and ON RGCs are impaired in mice with mild and sustained IOP elevation (Feng et al., 2013, 2016; Chen et al., 2015). Della Santina et al. (2013) reported that OFF transient RGCs are particularly vulnerable to IOP elevation, yet another study reported that SMI-32–labeled RGCs, which also include some OFF transient RGCs, are resistant to optic nerve crush injury (Duan et al., 2015). The absence of an agreed basis on which to classify mammalian RGCs (Sanes and Masland, 2015) and potential variation in RGC susceptibility to different optic neuropathies may account for some interstudy inconsistency. It is now claimed that there are >30 types of murine RGCs (Baden et al., 2016); thus classifications based solely on center response polarity (OFF, ON, ON-OFF) or response dynamics (sustained, transient) provide insufficient permutations to account for so many RGC subtypes. Thus, if susceptibility of different RGC subtypes to optic neuropathy is to be characterized well, other means of cell identification are needed.

Brain-derived neurotrophic factor (BDNF) plays important roles in neural development, survival, and function (Huang and Reichardt, 2003), as well as in neuroprotection during neurodegenerative disorders (Nagahara and Tuszynski, 2011). A series of studies has suggested that BDNF protects RGCs in experimental glaucoma (Johnson et al., 2009, 2011; Danesh-Meyer, 2011; Chader, 2012). Different approaches have been used to manipulate BDNF levels, such as intravitreal injection of BNDF protein, infection with adeno-associated virus expressing BDNF for gene therapy, and transplantation of stem cells in animal models of experimental glaucoma or optic neuropathy (Mey and Thanos, 1993; Chen and Weber, 2001; Martin et al., 2003; Harper et al., 2011). However, incomplete protection or insufficient rescue by BDNF were often observed in different models (Di Polo et al., 1998; Ko et al., 2001; Pease et al., 2009), and the mechanism underlying BDNF-mediated neuroprotection is not known.

We found recently that a modest increase of retinal BDNF protects some RGC subtypes in a mouse model of experimental glaucoma (Feng et al., 2016). Here we have investigated how BDNF affects RGC subtype survival in an acute model of optic nerve injury. As in our earlier study, we used a tamoxifen-induced Cre-ER system to up-regulate BDNF in the mouse retina (Feng et al., 2016). A rapid loss of RGCs was induced by crush of the optic nerve, and the effect of BDNF upregulation on RGC survival assessed by using a previously established *in vivo* imaging approach (Puyang et al., 2016).

## Materials and Methods

### Animals

BDNF-overexpression (BDNF_OE) mice were generated as described previously (Feng et al., 2016): BDNF^stop^ mice, which carry a stop cassette flanked by loxP inserted before the BDNF coding region, were crossed with Thy-1-CreER^T2^ transgenic mice that carry both enhanced YFP and CreER recombinase driven by the Thy-1 promoter (The Jackson Laboratory, stock no. 007606). One intraperitoneal injection of tamoxifen (10 mg/kg, dissolved in sunflower seed oil) was administered to turn on transcription of the BDNF transgene in adult Thy-1-CreER^T2^::BDNF^stop^ mice of either sex, thus creating BDNF_OE mice (Feng et al., 2016). All animal procedures were approved by the Northwestern University Institutional Animal Care and Use Committee and performed in accordance with the guidelines on the Use of Animals in Neuroscience Research from the National Institutes of Health and the Society for Neuroscience.

### Optic nerve crush surgery

The optic nerve crush (ONC) surgery was performed on left eyes only (Leung et al., 2011; Puyang et al., 2016). In brief, a small opening was created in the superior and lateral conjunctiva of the anesthetized mouse (Puyang et al., 2016). A pair of self-clamping forceps (#7, WPI) was used to clamp the optic nerve 0.5–1 mm behind the globe for 3 s (Puyang et al., 2016). Body temperature was maintained using a heating pad (Sunbeam) until mice were fully awake.

### *In vivo* imaging

*In vivo* images of retinas were captured via a Micron III fundus scope (Phoenix Research Laboratories; Puyang et al., 2016). Retinal images were taken before ONC (baseline) and at different time points after ONC. Images were then imported and processed by a customized Matlab program (The Mathworks). RGCs with soma diameter >21 µm were selected by the Matlab program, whose reliability was confirmed manually by two independent observers. We also compared the counting results from *in vivo* images with the soma counts from confocal images of the same retinas and found a strong positive correlation between the counts (*R* ^2^ = 0.89; Puyang et al., 2016). The survival rate of RGCs for each retina was expressed as the ratio of surviving RGCs to the total RGC number before ONC (baseline). The Kaplan–Meier estimator was used to give the probability of RGC survival (Puyang et al., 2016). A log-rank test was performed to compare survival distributions of different groups (Puyang et al., 2016).

### Immunohistochemistry and Western blot analysis

Whole-mounted retinas were prepared for immunostaining and retinal protein samples for Western blot analysis as described previously (Liu et al., 2007; Yoshida et al., 2011; Puyang et al., 2016; Feng et al., 2016). Primary antibodies for immunostaining included rabbit anti–green fluorescent protein (anti-GFP; 1:1000; Thermo Fisher Scientific, A-6455), mouse anti–Brn-3a (1:400; EMD Millipore, MAB1585), goat anti–Brn-3b (1:1000; Santa Cruz Biotechnology, sc-6026), mouse anti-TH (tyrosine hydroxylase, 1:400, EMD Millipore, MAB318), choline acetyltransferase (ChAT; 1:200; EMD Millipore, MAB305; Liu et al., 2007), BETA3 (also known as BhlhB5, 1:1000; Santa Cruz Biotechnology, sc-6045; Feng et al., 2006), cocaine- and amphetamine-regulated transcript (CART; 1:2500, Phoenix Pharmaceuticals), SMI-32 (neurofilament H non-phosphorylated, 1:1000, Covance; Feng et al., 2013), rabbit anti-BDNF (1:400; EMD Millipore, Ab1779SP; Feng et al., 2016), and mouse anti-GAPDH (1:2000, EMD Millipore, MAB374; Yoshida et al., 2011). Alexa Fluor–conjugated secondary antibodies were used (1:1000; Invitrogen), and images were captured with a Zeiss Pascal confocal microscope. Western blot analysis was performed using rabbit anti-BDNF (1:400; EMD Millipore, Ab1779SP) and mouse anti-GAPDH (1:2000, EMD Millipore, MAB374; Feng et al., 2016).

### Axon counts

Automatic axon counting was performed using a customized Matlab program as described previously (Puyang et al., 2016). Confocal images were taken of the flat-mounted retinal samples immunostained with GFP antibody. After positioning the center of the optic nerve head (ONH) in the image, the signal intensity at 200 and 400 μm radially surrounding the ONH was recorded, locating axons based on the local maxima (Puyang et al., 2016). After the contrast and brightness of the images were adjusted in Photoshop (Adobe Systems), two independent observers confirmed the number of axons manually.

## Results

### Effect of BDNF loss induced by ONC was compensated in BDNF_OE mice

**Figure 1.**
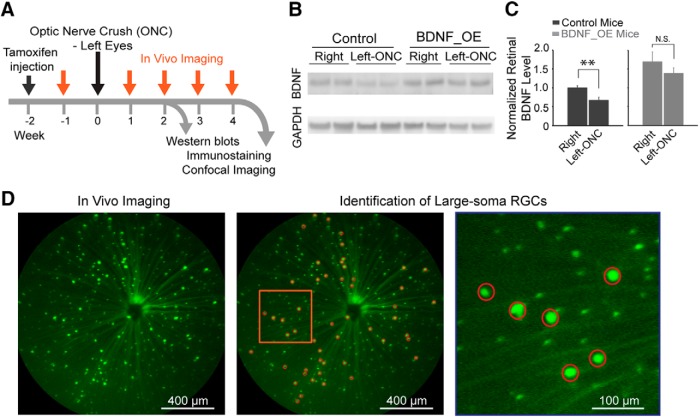
Experimental design to investigate the neuroprotective role of BDNF after ONC. ***A***, Timeline of experiments. ***B***, ***C***, Retinal BDNF levels were quantified by Western blot analysis. Left eyes received ONC, and relative protein levels were compared to the level of nonoperated right eyes (with right eyes of control mice designated as 1). BDNF_OE compensated for the down-regulation of retinal BDNF level induced by ONC. *n* = 4–7 mice in each group. N.S.: not significant; **p < 0.01 by Student’s *t*-test. ***D***, *In vivo* imaging to identify a small group of large-soma RGCs. Left, a representative Thy-1-CreER^T2^ mouse retina imaged by a Micron III fundus scope. Middle, the image was processed by Matlab to identify large-soma RGCs (marked by red circles). The area marked by the orange square was highlighted on the right.

We induced an acute loss of RGCs by ONC and determined whether overexpression of BDNF aided RGC survival (see schematic experimental design in Fig. 1*A*). We generated conditional BDNF-OE mice using the tamoxifen-induced Cre-recombinase system as described previously (Feng et al., 2016). ONC surgery was performed in left eyes, and the retinal BDNF levels were compared with the nonoperated right eyes of the same mice (Fig. 1*B*, *C*). At 2 weeks after the ONC injury, the BDNF level of left eyes from control mice was down-regulated 33% (0.67 ± 0.08) compared with the nonoperated right eyes (normalized as 1, *p* < 0.01 by Student’s *t*-test; Fig. 1*B*, *C*). In contrast, in BDNF_OE mice, the down-regulation of BDNF of left eyes induced by ONC injury was compensated for by BDNF overexpression (left, 1.70 ± 0.26 vs. right, 1.39 ± 0.15; *p* = 0.33 by Student’s *t*-test; Fig. 1*B*, *C*).

In this study, we used the Micron III fundus scope to track RGC loss after ONC injury (Feng et al., 2016). In the Thy-1-CreER^T2^::BDNF^stop^ mice, YFP-labeled somas of varying size were scattered across the retina within the ganglion cell layer (GCL; Fig. 1*D*, left). Because both RGCs and displaced amacrine cells are found in the GCL, we chose to focus on the effect of BDNF protection after ONC on one RGC subtype.

### Selection of large-soma RGCs

**Figure 2.**
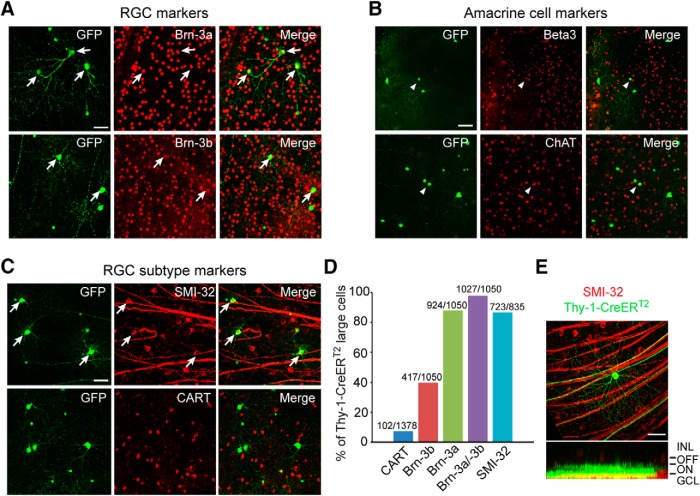
Identification of one type of RGCs with large somas. ***A***, GFP-positive cells with large somas expressing Thy-1-CreER^T2^ transgene were coimmunolabeled with RGC markers: Brn-3a and Brn-3b (arrows). ***B***, Small-soma, but not large-soma, GFP-positive cells were coimmunolabeled with amacrine cell markers: BETA3 and ChAT (arrowheads). ***C***, GFP-positive cells with large somas were coimmunolabeled with RGC subtype markers: SMI-32 (arrows), but not with CART. ***D***, Percentages of large-soma RGCs expressing Thy-1-CreER^T2^ transgene coimmunolabeled with different RGC markers. ***E***, Confocal image of one large-soma RGC double-labeled by SMI-32 (red) and GFP (green). Orthogonal view of the same cell is shown at the bottom. Most SMI-32 and GFP cohorts in large RGCs (20 of 28) had their dendrites laminated in ON sublamina of IPL. Scale bars: 50 µm. INL, inner nuclear layer.

Because amacrine cells generally have smaller somas than RGCs (Sun et al., 2002), we selected only cells with soma diameter >21 µm to exclude amacrine cells from this study (Fig. 1*D*, middle and right). To rule out the possibility that a number of amacrine cells may be included in the cell sample studied, we performed double-immunolabeling with amacrine cell marker ChAT, which labels starburst amacrine cells, and BETA3, which labels a small portion of displaced amacrine cells. We found that 0 of 343 ChAT-positive and of 179 BETA3-positive amacrine cells were colabeled with large-soma YFP-positive cells (Fig. 2*B*).

To confirm that the cells with large somas were RGCs, we used double-immunolabeling with the RGC markers Brn-3a and Brn-3b (Fig. 2*A*, *D*). We found that 98% of YFP-positive RGCs with large somas colabeled for Brn-3a (88%) or Brn-3b (40%; Fig. 2*A*, *D*). We further identified RGC subtypes using SMI-32, a marker for large alpha cells, and CART, a marker for ON-OFF direction-selective RGCs. Eighty-seven percent of large-soma RGCs colabeled for SMI-32, but only 7.4% for CART (Fig. 2*C*, *D*). In other words, most large-soma Thy-1–positive cells are SMI-32–positive RGCs. Moreover, these large-soma RGCs account for only 10.8% of the total SMI-32–positive cells (723 of 6723 SMI-32^+^ cells; *n* = 4 retinas).

To further identify whether these SMI-32–positive cells were ON or OFF RGCs, we examined their dendritic lamination pattern by confocal imaging. We identified 28 large-soma cells that were SMI-32– and YFP-positive and found that 20 of 28 cells were ON type, with dendrites in the ON sublamina of inner plexiform layer (IPL; Fig. 2*E*).

### Overexpression of BDNF delayed the loss of large-soma RGCs after ONC

**Figure 3.**
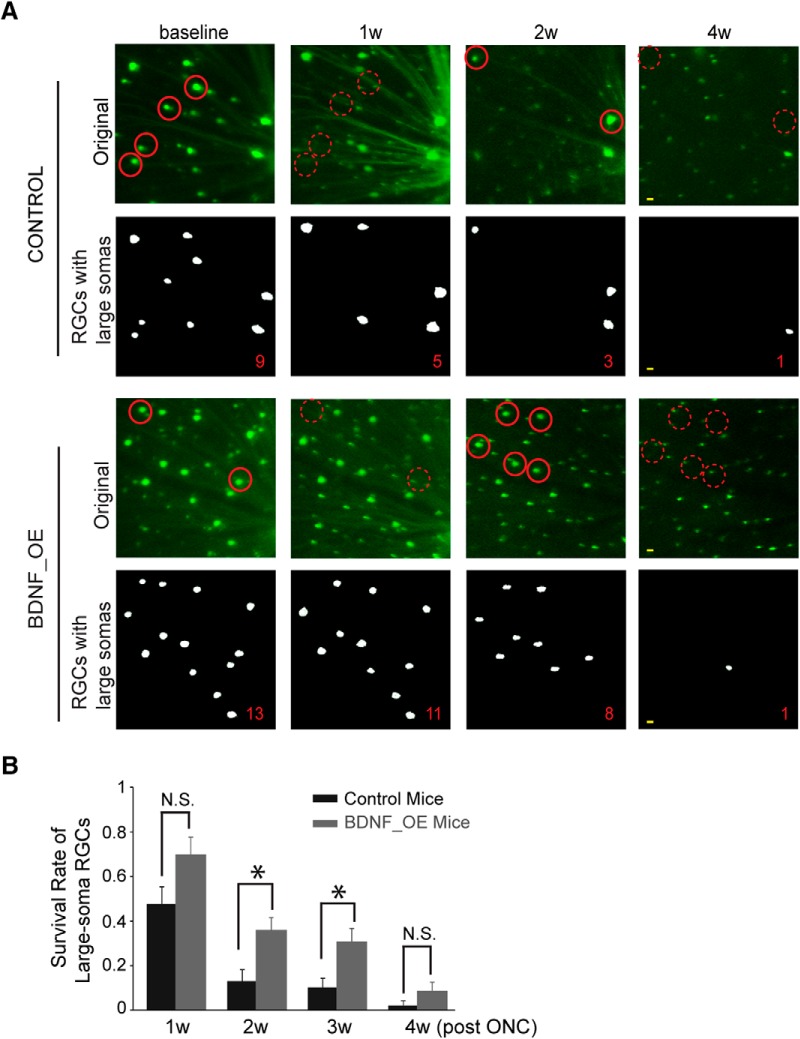
The loss of large-soma RGCs was delayed by BDNF overexpression. ***A***, *In vivo* and Matlab-processed images show the progressive loss of large-soma RGCs after ONC. Number of surviving RGCs is shown at the bottom right of each image. Some examples of the large-soma cells are circled in solid red lines, and the same locations the next week are circled in dashed lines, demonstrating the disappearance of these large somas. Scale bar: 20 µm. ***B***, More RGC somas survived in BDNF_OE than control mice. Control, *n* = 8 mice; BDNF_OE, *n* = 11 mice. N.S.: not significant; *p < 0.05 by Student’s *t*-test. Data are presented as mean ± SEM.

*In vivo* imaging was performed before ONC (baseline) and then every week for 4 weeks after ONC (Fig. 3*A*). RGCs with large somas were tracked for mice with and without BDNF upregulation. In the control mice, only 48% ± 8% of RGCs survived for 1 week after ONC (*n* = 8 mice, 228 cells; Fig. 3*B*). The RGC loss continued, with just 16% ± 5% remaining at 2 weeks and 10% ± 4% at 3 weeks (Fig. 3*B*). After 4 weeks, most, if not all, RGCs had died (2% ± 2%; Fig. 3*B*). In contrast, in the BDNF_OE mice, RGC survival rates were 70% ± 8% at 1 week (*n* = 11, 416 cells), a change not statistically significant from control mice (*p* = 0.06 by Student’s t-test; Fig. 3*B*). Many more cells survived at 2 weeks (36% ± 6%) and 3 weeks (31% ± 6%; Fig. 3*B*). However, by 4 weeks after ONC, BDNF_OE exhibited significant RGC loss (9% ± 4%) like the control group (Fig. 3*B*). Our data thus suggest that overexpression of BDNF delays RGC loss after ONC.

We used the Kaplan–Meier estimator to model the survival probabilities of RGCs for control and BDNF_OE mice. The median survival time for controls in this study is ∼7 d. For BDNF_OE mice, the median survival time of large-soma RGCs was ∼14 d, significantly longer than for control mice (*p* < 0.001 by log-rank test). In other words, overexpression of BDNF prolongs survival for large-soma RGCs by ∼1 week.

In addition, the median survival time for RGCs with large somas was 1 week less than we reported previously for median RGC survival in the Thy-1-YFP (H) mouse line. Although the time difference of the two transgenic lines failed to reach statistical significance (*p* = 0.06, two-way ANOVA), it indicates that large-soma RGCs may be among the most vulnerable to optic nerve trauma.

### Axon loss was also delayed in mice overexpressing BDNF after ONC injury

**Figure 4.**
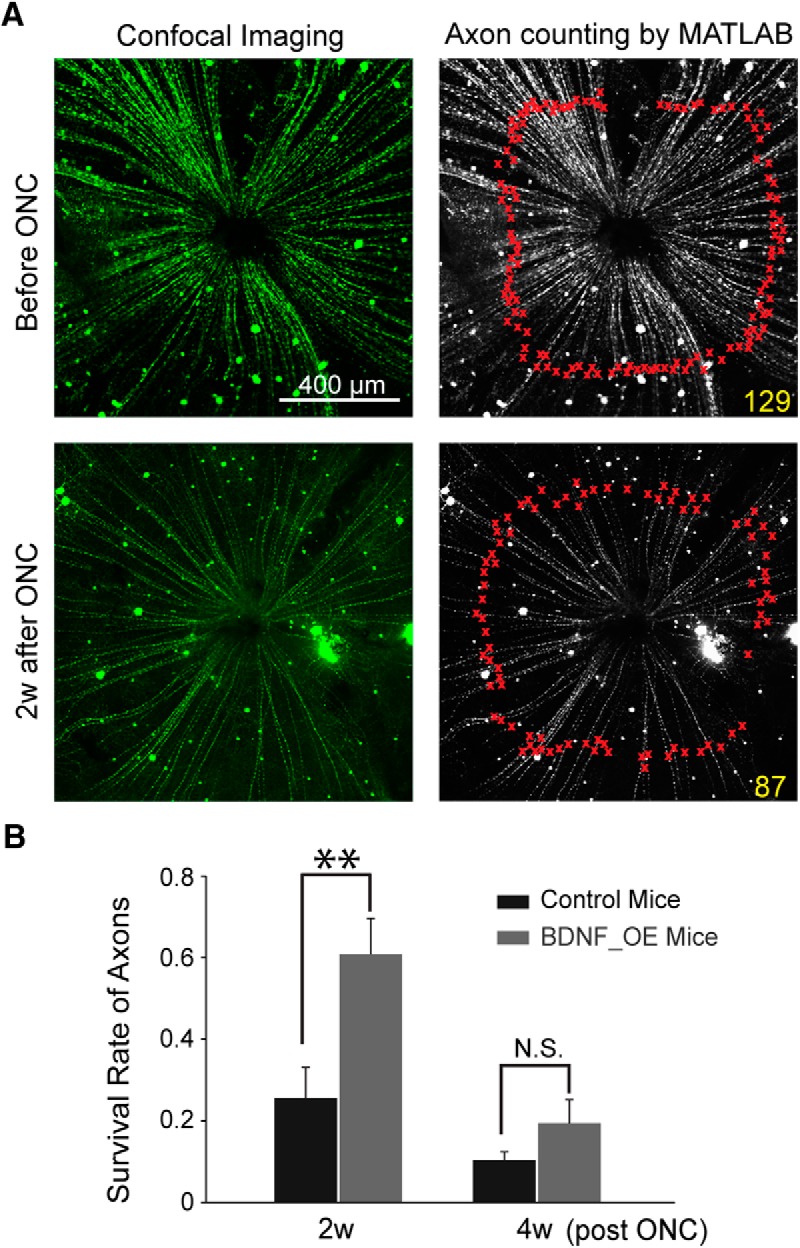
Axonal survival was also extended in BDNF_OE mice. ***A***, Confocal images of flat-mounted BDNF_OE retinas before and after ONC injury (left). Axons were immune-stained with anti-GFP antibody and counted with Matlab. Each red cross marks one axon by Matlab; the number was double-checked manually. ***B***, RGC survival was significantly higher in BNDF_OE mice at 2 weeks after ONC. *n* = 4–5 mice. N.S.: not significant; **p < 0.01 by two-way ANOVA, Sidak’s multiple comparison posttest. Data are presented as mean ± SEM.

We counted GFP-labeled axons from the entire retina with confocal imaging (Fig. 4*A*). Flat-mounted retinas were immunostained with anti-GFP antibody, and axon numbers were counted (Fig. 4*A*). Before ONC, the average axon number per retina was 167 ± 8 for control mice (*n* = 5; Fig. 4*A*). Approximately 26% ± 8% of axons survived 2 weeks after ONC (*n* = 4; Fig. 4*B*), a percentage consistent with our previous work (*p* = 0.33, Student’s *t*-test; Puyang et al., 2016). At 4 weeks after injury, only 10% ± 2% of axons survived in control mice (*n* = 4; Fig. 4*B*), demonstrating again that RGC loss after optic nerve injury is progressive and rapid. We found that axon survival was enhanced in the BDNF_OE mice (*p* < 0.01 by two-way ANOVA with Sidak’s multiple comparison posttest; Fig. 4*B*). At 2 weeks after the injury, 61 ± 9% of axons survived in BDNF_OE mice (102 ± 15 axons, *n* = 4 mice), much higher than for controls (*p* < 0.01 by two-way ANOVA with Sidak’s multiple comparison posttest; Fig. 4*B*). At 4 weeks, axonal survival in BNDF_OE mice was 19% ± 6% (*n* = 4 mice), twice the number for control mice, although the difference was not statistically significant (*p* > 0.05 by two-way ANOVA; Fig. 4*B*).

## Discussion

Recent studies suggest that the structural and functional degeneration of RGCs is type dependent in mouse models of experimental glaucoma or optic neuropathy, yet there are interstudy inconsistencies concerning the vulnerability of different types of RGCs (Feng et al., 2013; Della Santina et al., 2013; Chen et al., 2015; Duan et al., 2015; El-Danaf and Huberman, 2015). Considering that the median survival time for RGCs in Thy-1-YFP-H mice was 2 weeks (Puyang et al., 2016), that the median survival time for large-soma RGCs reported here (Fig. 3*C*) was just 1 week after ONC indicates that this RGC subpopulation is unusually vulnerable to optic nerve injury. This result contrasts with that of Duan et al. (2015), which showed genetically labeled RGCs (mostly SMI-32 positive) surviving longer than average. Because the SMI-32–positive large-soma RGCs account for only 11% of the total SMI-32 cells, our results suggest that SMI-32–positive cells may include different subgroups of RGCs, and each subgroup may respond to the insult differently.

There is also some disagreement among investigators about the vulnerability of ON RGCs cells to optic nerve injury or glaucomatous insult. We have found some SMI-32 ON cells to be more vulnerable to sustained IOP elevation (Feng et al., 2013), whereas another study reported that the ON layer of the IPL was unperturbed at an early stage of IOP elevation (El-Danaf and Huberman, 2015). Physiological studies of mouse RGCs have shown that ON cells have smaller receptive field centers after 5–7 weeks of IOP elevation (Chen et al., 2015) and lower spontaneous rates and light-evoked responses (Della Santina et al., 2013). In this study, we followed a small number of SMI-32–positive RGCs, most of which were ON cells (71%, Fig. 2*E*). We found that these cells were unusually susceptible to ONC (Fig. 3). The weight of results therefore supports that these large-soma ON RGCs are more vulnerable to optic nerve insults than other RGC varieties. Further studies are needed to better classify individual RGC types to understand the mechanisms underlying the type-specific RGC loss in different animal models.

Furthermore, we employed *in vivo* imaging to follow the same RGCs over time, avoiding intersample variation after ONC (Leung et al., 2011; Feng and Liu, 2016; Puyang et al., 2016). Leung et al. (2011) applied a blue-light confocal scanning laser ophthalmoscope to track RGC survival *in vivo* using Thy-1-YFP-16Jrs transgenic mice. Puyang et al. (2016) used Thy-1-YFP-H mice and showed that the overall RGC survival rate decreased progressively after injury. We further confirmed that the overall survival of axons labeled by Thy-1-YFP-H and Thy-1-YFP-CreER^T2^ exhibited no significant difference 2 weeks after ONC (Fig. 4*B*; current study and Puyang et al., 2016), suggesting that both transgenic lines provided consistent sampling of all RGCs and their axons. One encouraging future direction would be to combine *in vivo* imaging and transgenic mouse lines with specific RGC subtypes labeled to study RGC subtype loss at a fine granularity after disease insult.

We found that the survival of axons increased ∼1.38-fold by BDNF overexpression at 2 weeks after optic nerve injury (Fig. 4*B*). This increase is likely due to the increase of the survival rate observed in large-soma RGCs at the same stage (1.29-fold; Fig. 3*B*). Our studies suggest that BDNF encouraged the survival of large-soma RGCs after acute optic nerve injury. During normal development, type-specific regulation led by neurotrophin-3 and BDNF is important for the maturation of RGCs (Liu et al., 2007, 2009). In diseased conditions, the type-specific protection may explain the partial or incomplete neuroprotective effects observed in previous studies (Mey and Thanos, 1993; Chen and Weber, 2001; Weber et al., 2010; Feng et al., 2016). In addition, neuroprotective agents have targeted the multiple pathogenic mechanisms that result in axonal degeneration and RGC death, which include anti-apoptotic strategies, tumor necrosis factor-α, anti-excitotoxic agents, and stem cells (Danesh-Meyer, 2011). The differential vulnerabilities of RGC subtypes are likely induced by diverse signaling pathways, and these different pathways may interact to activate apoptotic effects, which might explain that by the end of 4 weeks after ONC, the majority of RGCs were still lost in BDNF_OE retina.

Together, we find that (1) large-soma RGCs (∼11% of SMI-32–positive cells) are particularly susceptible to optic nerve injury; and (2) overexpression of BDNF prolongs the survival of RGCs, including large-soma RGCs.

## Synthesis

Reviewing Editor: Leonard Maler, University of Ottawa Decisions are customarily a result of the Reviewing Editor and the peer reviewers coming together and discussing their recommendations until a consensus is reached. When revisions are invited, a fact-based synthesis statement explaining their decision and outlining what is needed to prepare a revision will be listed below. The following reviewer(s) agreed to reveal their identity: Zhigang He

Editor note

Many of the requested changes to the original Journal of Neuroscience submission have been successfully carried out. There are still substantial changes required as described in detail below.

And, it is most important that the authors demonstrate, in the Significance statement as well as in the Introduction and/or Discussion, how this Ms substantially extends our current knowledge in this field as per: “...the main conclusion of the current study appears to be limited.”

Reviewer #2

Previous studies have demonstrated that BDNF increases RGCs survival in experimental models of optic neuropathies (Di Polo et al., 1998; Feng et al., 2016; Zhang et al., 2005). The fact that RGCs continue to die after optic nerve injury in animals with increased BDNF expression may suggest that other pro-survival factors are lacking. Detrimental changes in the extracellular environment also impact on neuron survival after injury. Di Polo et al. suggested that “the effects of BDNF on RGC survival is limited by changes induced by the lesion within 10-16 days after optic nerve transection rather than the availability of BDNF”. Thus, it is unlikely that BDNF alone would preserve injured RGCs for long period of time (e.g. more than 2 weeks) after optic nerve injury. In light of this evidence, the main conclusion of the current study appears to be limited. In addition, it is not clear why the authors decided to focus on a particular subclass instead of looking at the fate of different subclasses of RGCs.

Figure 1C: A 1-way ANOVA with Dunnet posttest may be more appropriate here.

Figure 3B. The authors should consider using a multiple regression model (Cox regression, log-rank test) and not a t-test for data analysis

The protective effect of neurotrophic factors such as brain derived neurotrophic factor (BDNF) makes them promising therapeutic candidates for neurodegenerative diseases including glaucoma.

By utilizing a longitudinal in vivo imaging approach combined with a mouse genetic strategy, the authors of the current study investigated the effects of a modest increase of BDNF on RGCs survival after optic nerve injury in mice. In particular, the authors focused on a subclass of SMI-32 positive RGCs with large soma that are susceptible to optic nerve injury. Increasing BDNF levels was sufficient to extend survival of these neurons after injury.

Overall, the experiments are well executed and the longitudinal imaging approach is establishing a new state of the art for the field. The conclusion that a modest increase of BDNF protects a subclass of RGCs, namely large-soma SMI-32 positive, is supported by the results.

Major concern:

Previous studies have demonstrated that BDNF increases RGCs survival in experimental models of optic neuropathies (Di Polo et al., 1998; Feng et al., 2016; Zhang et al., 2005). The fact that RGCs continue to die after optic nerve injury in animals with increased BDNF expression may suggest that other pro-survival factors are lacking. Detrimental changes in the extracellular environment also impact on neuron survival after injury. Di Polo et al. suggested that “the effects of BDNF on RGC survival is limited by changes induced by the lesion within 10-16 days after optic nerve transection rather than the availability of BDNF”. Thus, it is unlikely that BDNF alone would preserve injured RGCs for long period of time (e.g. more than 2 weeks) after optic nerve injury. In light of this evidence, the main conclusion of the current study appears to be limited. In addition, it is not clear why the authors decided to focus on a particular subclass instead of looking at the fate of different subclasses of RGCs. In my opinion, the authors should address this knowledge gap.

Minor concerns:

1. Inconsistencies between studies may stem from differences in injury type, location, and severity. For clarity and reproducibility, it is important referring to the same injury model by using the same terminology. The authors stated that they have used a “partial optic nerve crush” (Page 5, line 102), while Duan et al. used a “complete optic nerve axotomy” (Duan et al., 2015) (Page 3, line 58). To my understanding (for details, refer to the Material and Methods section of Duan et al., 2015), both studies used the same injury paradigm, more commonly referred as “optic nerve crush” (ONC) model. To avoid any confusion, I would suggest changing the text accordingly and unifying the terminology.

2. In Figure 1D, the box in the centered panel is hard to see. The authors may consider changing the color to increase the contrast with the image background.

3. Figure 1C: A 1-way ANOVA with Dunnet posttest may be more appropriate here.

4. Figure 2. The authors may consider reducing the scale bar to 50 microns to facilitate comparison with the neuronal cell bodies.

5. Figure 3B. The authors should consider using a multiple regression model (Cox regression, log-rank test) and not a t-test for data analysis.
